# Effect of RTOG breast/chest wall guidelines on dose‐volume histogram parameters^*^

**DOI:** 10.1120/jacmp.v15i2.4547

**Published:** 2014-03-06

**Authors:** Sonali Rudra, Hania A. Al‐Hallaq, Christine Feng, Steven J. Chmura, Yasmin Hasan

**Affiliations:** ^1^ Department of Radiation Oncology Georgetown University Hospital Washington D.C. 20007 USA; ^2^ Department of Radiation and Cellular Oncology The University of Chicago Medical Center Chicago IL USA

**Keywords:** dose‐volume histogram, RTOG, breast contouring, chest wall contouring

## Abstract

Treatment planning for breast cancer has been traditionally based on clinical landmarks. The Radiation Therapy Oncology Group (RTOG) published consensus guidelines on contouring target volumes (TV) for the breast/chest wall and draining lymphatics. The effect of these guidelines on dosimetric parameters in surrounding organs at risk (OAR) and TVs is unknown. Fourteen patients treated with clinically derived plans from 2007‐2011 (Group I) and fourteen patients treated with target volume‐based plans from 2011‐2012 were selected for comparison (Group II). Treatment plans were constructed based on clinical landmarks (Group I) or TVs (Group II) to a median dose of 50.4 Gy to the breast/chest wall, axilla (Ax), supraclavicular (SCV), and internal mammary (IMN) lymph nodes. The RTOG TVs were then contoured in Group I patients by a single investigator blinded to the dose distributions. Dose‐volume histograms (DVH) were computed for the RTOG TVs and OARs in both groups, and DVH parameters were compared. In Group II, coverage improved for the SCV (V90=78.0% versus 93.6%, p=0.02) and intact breast (V95=95.6% versus 99.3%, p=0.007). The dose to the cord, the lung (V20Gy and V30Gy), and contralateral breast (V5Gy) were the same. Finally, the low dose to the heart and lung was decreased in Group II (heart V5Gy=48.7% versus 27.3%, p=0.02, heart V10Gy=33.5% vs. 17.5%, p=0.01, and ipsilateral lung V5Gy=84.5% vs. 69.3%, p=0.001). Overall, our study supports that treatment planning using the RTOG consensus guidelines can improve coverage to certain target volumes compared to treatments based solely on clinical landmarks. Additionally, treatment planning using these target volumes does not increase dose to the contralateral breast, cord, heart, or lungs. Longer follow‐up is needed to determine if using these target volumes will affect clinical outcomes.

PACS number: 87.55.dk

## I. INTRODUCTION

Traditionally, breast cancer patients have been treated with conventional fields based on marks placed around the breast/chest wall to delineate targets based on bony/anatomical landmarks at the time of simulation. In the modern era of 3D CT‐based planning, treatment planning for breast cancer patients is evolving. Most institutions are using CT simulation scans to modify the placement of the treatment fields, and many are beginning to use target volumes for treatment planning for either 3D‐conformal or IMRT treatments. This shift is supported by two randomized studies of IMRT and volume‐based planning in breast cancer that demonstrated improvement in moist desquamation and cosmetic outcomes with volume‐based planning compared to 2D planning.^1^, ^2^

Concurrently, the evidence and clinical indications for regional nodal irradiation both after mastectomy and breast‐conservation surgery have become better established. Previously, the consensus guidelines suggested that women should receive postmastecomy radiation to the chest wall and regional nodes if they had > 4 involved lymph nodes, tumors >5 cm, or a combination of multiple intermediate risk factors, based primarily on the British Columbia and Danish randomized trials.^3^, ^4^, ^5^, ^6^ However, the long‐term follow‐up of the Danish trial has shown a locoregional control and survival benefit of postmastecomy chest wall and regional nodal irradiation in patients with one to three lymph nodes involved, as well as patients with > 4 lymph nodes involved.^(7)^ Additionally, recent data from the NCIC MA.20 trial, which analyzed the utility of regional nodal irradiation after breast‐conserving surgery, showed an improvement in locoregional disease‐free survival (DFS), distant DFS, and overall DFS for women with an intact breast.^(8)^

Given these recent changes, the Radiation Therapy Oncology Group (RTOG) published consensus guidelines to contour the target volumes (TV) for the breast, chest wall, and draining lymphatics.^(9)^ These guidelines were created to address the variability when contouring these structures between different physicians.^(10)^ The practical outcome for using these target volumes to develop treatment plans remains unclear, specifically in terms of the effects of dose to the surrounding normal structures and whether the dose to the proposed TVs differs from dose received when using clinically‐guided treatment plans.

In this analysis, we evaluate the differences in dosimetric parameters to the organs at risk and target volumes in patients treated with clinically‐guided treatment plans compared to patients who were treated with target volume‐based treatment plans. We also assessed differences in acute toxicity within the two groups.

## II. MATERIALS AND METHODS

### A. Patient identification

Fourteen consecutive patients who were treated from 2011‐2012 with target volume‐based plans to the breast or chest wall and draining lymphatics, including the axilla (Ax), supraclavicular (SCV), and ipsilateral internal mammary (IMN) lymph nodes were selected. In these 14 patients (Group II), the RTOG target volumes and organs at risk (OARs) were contoured on the simulation CT scan. The target volumes included the breast or chest wall, Ax level I, Ax level II, Ax level III, SCV, and IMN lymph nodes. While we currently specifically target the full axilla in patients who have N2/N3 disease or those with an incomplete dissection, at the time the patients in this analysis were treated, our institutional practice was to treat patients with node positive disease and other high risk features with comprehensive nodal irradiation, as per the randomized data.^3^, ^4^ Target volumes for the chest wall or breast were delineated based on radiopaque markers placed at the time of simulation. The OARs included the heart, ipsilateral lung, whole lung, contralateral breast, and spinal cord. All contours of the target volumes and OARs were approved by one of two breast radiation oncologists. Treatment plans were then constructed to treat the target volumes to a median dose of 50.4 Gy to the breast/chest wall, SCV, Ax, and IMN lymph nodes. The volumes were not modified at the time of treatment planning.

As a comparison group, fourteen additional patients (Group I) who were planned prior to the use of the RTOG target volumes from 2007‐2011 were selected to match the type of treatment received (radiation to the chest wall versus intact breast) and tumor laterality (left versus right sided tumors) of the Group II patients. All of the Group I patients were also treated to the breast or chest wall, full axilla, and ipsilateral IMN. In Group I patients, radiopaque wires were placed at the time of CT simulation to define the field edges for the chest wall or intact breast. Bony landmarks were used to identify the SCV, IMN, and axillary lymph nodes, and the OARs were contoured by a dedicated breast dosimetrist. A treatment plan was developed based on these landmarks without routine contouring of the targets. Plans were optimized based solely upon visual inspection of dose in all axial views by one of the two breast radiation oncologists. The RTOG target volumes were then contoured in a manner consistent with the contouring for Group II for these 14 patients in Group I by a single physician investigator (SR) blinded to the dose distributions and used for comparison of the DVH parameters of these volumes with those of the Group II patients.

### B. Treatment planning

A commercial treatment planning system, Pinnacle (Version 9; Philips Medical System, Milpitas, CA) was used to generate all treatment plans. The chest wall or intact breast was treated with tangential fields with matching SCV and IMN fields using a mono‐isocentric beam arrangement. The SCV field was treated with an anterior oblique field with a posterior field when needed. A posterior field was used in all of the patients in Group II and 79.0% of patients in Group I to cover deep nodal target volumes and to reduce hotspots greater than 110.0%. The posterior beam was planned using CT‐based planning rather than prescribed to a depth, thereby ensuring adequate coverage without creating hotspots. The IMN lymph nodes were either included in the tangential fields or treated with an en‐face electron beam that was matched to the tangential fields on the skin. In general, the tangential fields included a maximum of 2‐3 cm of lung tissue to cover the breast or chest wall tissue. None of the patients in this analysis were treated with an inverse planned IMRT technique. All treatment plans were modified based on patient and tumor factors and approved by a radiation oncologist prior to treatment. The treatment plans were also reviewed and approved at the weekly departmental QA meetings. The median dose to the target volumes was 50.4 Gy in 1.8 Gy fractions (46.8‐50.4 Gy). Some patients also received an additional boost at the physician's discretion to either the lumpectomy cavity or chest wall to a median dose of 10 Gy (8‐16 Gy). Conventional photon beams of 6 MV and 18 MV, if necessary, were used for planning, and all calculations used tissue heterogeneity corrections.

### C. Dose‐volume histogram

Dose‐volume histograms (DVH) were computed for the RTOG target volumes and OARs in both groups. During treatment planning for Group I patients, the primary dose constraint was to cover 95.0% of the target with 95.0% of the prescribed dose for the breast or chest wall, per RTOG guidelines. The dose constraint for the IMN was to cover 100.0% of the volume by 80.0% of the dose. For Group II patients, treatment plans were prospectively constructed based on institutional DVH constraints for the target volumes, which are listed in Table 1. The dose constraints for the normal organs were the same for both Group I and Group II patients and are also found in Table 1. These dosimetric parameters were recorded for all patients. We also retrospectively assessed additional parameters that were not usually assessed during treatment planning (esophageal V5Gy, V10Gy, V20Gy, V30Gy, ipsilateral and whole lung V5Gy and V10Gy, and heart V10Gy, V30Gy, and mean heart dose) to further determine if there were differences between the two planning methods.

**Table 1:** Dose constraints for breast/chest wall target volumes and organs at risk

*Organ*	*Constraint*
Chest Wall	V90^a^ ≥90.0%
Breast	V100≥90.0%
	V95≥95.0%
	V105≤40.0%
	V110≤10.0%
IMN Nodes	V80≥100.0%
SCV	V90≥90.0%
Ax Nodes	V90≥90.0%
Contralateral Breast	V5Gy^b^ ≤ 15.0%
Ipsilateral Lung	V20Gy≤45.0%
	V30Gy≤35.0%
Whole Lung	V20Gy≤25.0%
	V30Gy≤20.0%
	Heart V5Gy≤40.0% (≤50.0% for left‐sided tumors)
	V20Gy≤20.0%

^a^ Vx refers to the volume of the target volume receiving x% of the dose (i.e, V90≥90.0% means that greater than 90.0% of the volume was covered by 90.0% of the prescription dose).

^b^ VxGy refers to the volume of the target volume receiving × Gy (i.e., V5Gy≤15.0% means that less than 15.0% of the volume was covered by 5 Gy).

IMN=internal mammary lymph nodes; SCV=supraclavicular lymph nodes; Ax=axillary lymph nodes.

### D. Treatment outcomes

The patient charts were also retrospectively reviewed to assess the acute toxicities from treatment. Given that the Group II patients were treated in 2011‐2012, there is not sufficient follow‐up to report on long‐term toxicity. Clinical outcomes, such as local control, regional control, and overall survival, were also not assessed due to limited follow‐up. Review of patient data was approved by the Institutional Review Board protocol #15837A.

### E. Statistical analysis

Baseline patient, tumor, and treatment characteristics were compared with either a chi‐squared test for categorical variables or a Student's t‐test for continuous variables (JMP‐version 9; Cary, NC). The mean DVH parameters were compared using a Student's *t*‐test. DVH parameters were compared for the initial plans to 46.8‐50.4 Gy for the primary analysis. A comparison of the DVH parameters for the composite plans, including the boost, was also performed, when available.

## III. RESULTS

### A. Patient characteristics

Patients and treatment characteristics for the two groups are listed in Table 2.

**Table 2:** Patient and treatment characteristics

*Characteristic*	*Group I (Clinically‐Guided Plans)*	*Group II (Target Volume‐based Plans)*
Median Age	53	48, p=0.25
Range	40−68	33−77
Stage		
IB	1	0
IIA	1	1
IIB	1	2
IIIA	4	7
IIIB	1	1
IIIC	4	2
N+	2	1
Chest Wall	11	11
Breast	3	3
Location		
Upper outer quadrant	3	11
Lower outer quadrant	4	1
Upper inner quadrant	4	0
inner quadrant	0	0
Central	1	1
Nodal	2	1
Left	8	8
Right	6	6
Separation	24.0 cm	24.2 cm, p=0.91
Body Mass Index	29.1	29.0, p=0.97
Dose		
Median breast/chest wall dose (range)	50.4 Gy (46.8Gy−50.4Gy)	50.4 Gy (50−50.4Gy)
Median boost dose (range)	12 Gy (10−16.2Gy)	10 Gy (10−16Gy)
Boost		
Chest wall boost	2	9
Lumpectomy boost	3	3
No Boost	9	2, p=0.007
IMN Treatment Technique		
Wide tangents	1	3
En‐face electrons	13	11, p=0.27
Neoadjuvant Chemo		
Yes	5	8
No	9	6, p=0.25
Cardiotoxic Chemotherapy	64.0%	78.0%, p=0.40
Herceptin use	14.0%	21.0%
Adriamycin use	50.0%	57.0%
Contralateral breast RT (previous or concurrent)	1	1

### B. comparison of DVH for target volumes

**Figure 1:**
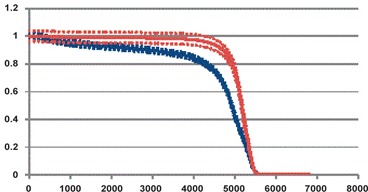
Composite SCV DVH of patients in Group I (blue) ± one standard deviation (blue dotted lines) compared to the composite SCV DVH of patients in Group II (red) ± one standard deviation (red dotted lines). Mean V90=78.0% (Group I) versus 93.6% (Group II) (p=0.02).

The coverage of target volumes was similar for most parameters analyzed between the two groups (Table 3). When the target volumes were contoured prior to treatment planning (Group II), there was better coverage of the breast (V95=95.6% vs. 99.3%, p=0.007). The supraclavicular coverage (V90) was also improved 78.0% versus 93.6% (p=0.02). In 71.0% of the Group I patients, the SCV region did not meet the dose constraint of V90≥90.0%. In the ten patients who did not receive adequate coverage, there was not sufficient coverage superiorly in two patients, medially in six patients, and both superiorly and medially in two patients. Figure 1 shows the SCV DVH (composite± one standard deviation) for Group I compared to Group II. The composite DVH was generated by averaging the dose for each volume increment across the 14 patients. The standard deviation of the dose for each volume increment was also generated and plotted on the same curve.

**Table 3:** Mean coverage of target volumes and OARs

*Structure*	*Group I Clinically‐derived (mean)*	*Group II Target Volume‐based (mean)*	*p‐value*
Breast V100^a^	90.8	91.9	0.71
Breast V95	95.6	99.3	0.007^b^
Breast V105	56.2	43.2	0.40
Breast V110	22.6	8.4	0.19
CW (V90)	94.4	96.2	0.21
IMN (80%)	99.0	96.4	0.18
SCV (V90)	78.0	93.6	0.02^b^
Ax^c^ 1 (V90)	96.6	98.7	0.12
A×2 (V90)	100	99.9	0.55
A×3 (V90)	100	99.9	0.51
Cord Max (Gy)	8.0	11.5	0.09
Ipsilateral Lung V5Gy^b^	84.5	69.3	0.001
Ipsilateral Lung V10Gy	63.9	57.5	0.19
Ipsilateral Lung V20Gy	44.5	44.1	0.92
Ipsilateral Lung V30Gy	34.1	37.1	0.23
Ipsilateral Mean Lung (Gy)	22.2	22.9	0.56
Whole Lung V5Gy	44.0	35.7	0.03^b^
Whole Lung V10Gy	33.2	29.4	0.28
Whole Lung V20Gy	23.4	22.6	0.74
Whole Lung V30Gy	17.8	18.9	0.55
Mean Whole Lung (Gy)	11.7	11.9	0.86
Heart V5Gy	48.7	27.3	0.02^b^
Heart V10Gy	33.5	17.5	0.01^b^
Heart V20Gy	11.3	8.1	0.24
Heart V30Gy	5.1	4.7	0.82
Mean Heart Dose (Gy)	9.0 Gy	6.4 Gy	0.08
Esophageal V5Gy	34.0	20.5	0.15
Esophageal V10Gy	14.3	13.5	0.86
Esophageal V20Gy	6.2	8.0	0.53
Esophageal V30Gy	3.0	5.2	0.28
Contralateral Breast V5Gy	2.7	1.7	0.36

^a^ Vx refers to the volume of the target volume receiving x% of the dose (i.e., V100 refers to the volume receiving 100.0% of the prescription dose).

^b^ The comparisons that were statistically significant.

^c^ Ax1 means axillary lymph nodes, level 1.

^d^ VxGy refers to the volume of the target volume receiving × Gy (i.e., V5Gy refers to the volume of the organ at risk receiving 5 Gy).

CW=chest wall; IMN=internal mammary lymph nodes; SCV=supraclavicular lymph nodes.

### C. Organs at risk

**Figure 2:**
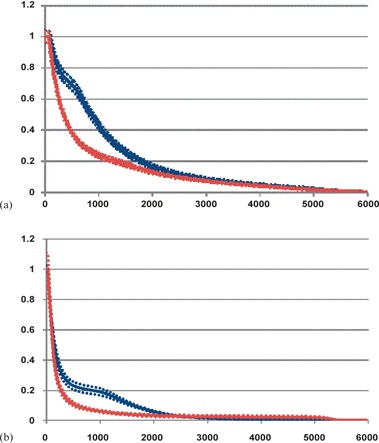
Composite heart DVH (a) of patients with left‐sided tumors in Group I (blue) ± one standard deviation (blue dotted lines) compared to patients with left‐sided tumors in Group II (red) ± one standard deviation (red dotted lines). Mean V5Gy=68.6% (Group I) versus 39.1% (Group II) (p=0.0004). Composite heart DVH (b) of patients with right‐sided tumors in Group I (blue) ± one standard deviation (blue dotted lines) compared to patients with right‐sided tumors in Group II (red) ± one standard deviation (red dotted lines). Mean V5Gy=22.2% (Group I) versus 11.5% (Group II) (p=0.10) and mean V20Gy = 5.9% (Group I) vs. 2.0% (Group II) (p=0.04).

In this analysis, the dose to the surrounding critical structures was not increased by using target volume‐based planning. The parameters for the ipsilateral and whole lung showed no difference between the two groups, except for the low dose (V5Gy=84.5% versus 69.3%, p=0.001 for the ipsilateral lung and V5Gy=44.0% versus 35.7%, p=0.03 for the whole lung) (Table 3). The low dose to the esophagus was assessed in the two groups and there was no difference in the parameters assessed. There was also no difference in dose to the contralateral breast (V5Gy=2.7% versus 1.7%, p=0.36) or the maximum spinal cord dose (8.0 Gy versus 11.5 Gy, p=0.09). Incidentally, there was a decrease in the low dose to the heart in Group II patients with mean V5Gy of 48.7% versus 27.3% (p=0.02) and mean V10Gy=33.5% versus 17.5% (p=0.01), but no difference in the V20Gy of 11.3% versus 8.1% (p=0.24). This difference was seen primarily for left‐sided tumors when left‐ and right‐sided tumors were analyzed separately. For left‐sided tumors, the V5Gy was 68.6% versus 39.1% (p=0.0004) and for right‐sided tumors, the V5Gy was 22.2% versus 11.5% (p=0.10). For right‐sided tumors, there was a difference in the mean heart V20Gy: 5.9% vs. 2.0% (p=0.04). The heart DVH (composite ± one standard deviation) is shown in Fig. 2.

### D. DVH for patients with boost

The DVH parameters for patients who received a chest wall boost were calculated using the composite plans, when available. Patients who underwent a boost to the lumpectomy cavity were treated with en‐face electrons in a treatment position that was different than the original; therefore, a composite plan was not generated for these patients. Only two patients in Group I had a composite plan, while eight patients in Group II had a composite plan. The mean values for the DVH parameters are listed in Table 4. Although patient numbers are small, it appears that even with the addition of the boost, there was not a large increase in the dose to the lungs in Group II patients compared to Group I patients who were treated with a boost. Additionally, we continue to see an improvement in the dose to the heart (V5Gy).

**Table 4:** Comparison of DVH parameters for OARs in patients receiving a chest wall boost

*Parameter*	*Group I* (n=2) *Mean Value*	*Group II* (n=8) *Mean Value*	*p‐value*
Ipsilateral Lung V20Gy^a^	39.8	43.5	0.39
Ipsilateral Lung V30Gy	31.0	36.5	0.10
Whole Lung V20Gy	21.1	22.2	0.71
Whole Lung V30Gy	16.3	18.6	0.33
Heart V5Gy	52.0	27.0	0.006
Heart V20Gy	13.6	7.8	0.13
Contralateral Breast V5Gy	0.79	1.8	0.26

^a^ VxGy refers to the volume of the target volume receiving × Gy (i.e., V20Gy refers to the volume of the organ at risk receiving 20 Gy).

### E. Acute toxicity

All patients developed acute dermatitis. In Group I, 57.0% developed Grade 1, 36.0% had Grade 2, and 7.0% had Grade 3 toxicity. In Group II, 50.0% had Grade 1, 43.0% had Grade 2, and 7.0% had Grade 3 toxicity (p=0.92). Patients in Group I had a higher incidence of fatigue (50.0% versus 21.0%, p=0.11) than in Group II, but this difference was not statistically significant. Only one patient developed a chronic cough of unknown etiology in Group II, and one patient developed esophagitis requiring viscous lidocaine in Group I.

## III. DISCUSSION

In our analysis, we found that routine contouring of the RTOG target volumes for breast cancer treatment planning allowed for good coverage of the target volumes without increasing the dose to surrounding structures. With the targets delineated, coverage was improved for the SCV (V90=78.0% versus 93.6%, p=0.02) and intact breast V95 (95.6% versus 99.3%, p=0.007). In terms of the surrounding normal tissues, the dose to the lungs was the same in both groups, except for the low dose (V5Gy) and the low dose to the heart was decreased (V5Gy and V10Gy). Although the comparison between patients in both groups who received a boost was limited, the addition of the boost also did not seem to increase dose to the normal structures.

The absolute difference in dose coverage was most improved in the supraclavicular region. Treatment of this region requires a cord block for the anterior oblique beam and it is possible that the block was placed in a manner that compromised coverage of the SCV region, especially since most patients were underdosed in the medial aspect of the SCV. In Group II, when the location of the SCV target volume was known, it is possible that the cord block was modified to improve coverage of the region. Adequate coverage of this region does increase the cord dose, although this difference was not statistically significant. Additionally, the maximum cord dose in Group II is still below the normal dose constraints of the spinal cord. The dose to the esophagus was also assessed and found to be comparable between the two groups.

The cause for the decrease in dose to the heart, in terms of V5Gy (48.7% versus 27.3%, p=0.02) and V10Gy (33.5% versus 17.5%, p=0.01) was assessed by analyzing potential differences in baseline tumor and treatment characteristics. Patients were matched in this study based on percentage of left‐sided tumors (57.0% in each group) and postmastectomy treatments (79.0% in each group). Therefore, a difference in number of left‐sided tumors or postmastectomy patients did not explain the difference in cardiac dose. There was a nonstatistically significant difference in the treatment technique to cover the IMN lymph nodes, with more patients being treated with an electron field matched to tangential fields in Group I (3/14 patients versus 1/14 patients). However, the mean heart V5Gy, V10Gy, V20Gy, and V30Gy doses were compared and not found to be statistically different (mean V5Gy=39.2% with electron/photon match versus 30.6% with wide tangents, p=0.53, mean V10Gy=25.5% vs. 25.4%, p=0.99, mean V20Gy=10.1% with electron/photon match versus 7.2% with wide tangents, p=0.47, and mean V30Gy=4.7% vs. 6.5%, p=0.53). It is possible that since patients in Group II were younger (median age 48 years versus 53 years, p=0.25) and had treatment with cardiotoxic chemotherapy more often (78.0% versus 64.0%, p=0.40), the attending physicians may have modified the angles or blocks to minimize the dose to the heart. However, the differences in age and cardiotoxic chemotherapy use were not statistically significant. We also assessed the heart volume in the two groups and found that, while the total heart volume was higher in Group I (638.8 cc versus 589.3 cc, p=0.36), this difference was not statistically significant. There was a difference in the location of the tumor between the two groups — only 50.0% of patients in Group I had outer quadrant tumors, while 79.0% of Group II patients had outer quadrant tumors (p=0.009). It is possible that breast tissue or chest wall coverage for the outer quadrant tumors required the tangential fields to encompass less of the normal cardiac tissue, leading to the decreased V5Gy and V10Gy to the heart. Another possibility for the difference is that contouring the target volumes enabled the physicians and dosimetrists to recognize the areas at risk and better cover those areas, while minimizing the dose to the heart. It is important to note that the heart volume was contoured prior to treatment planning in both groups. While there were differences in the use of boost between the two groups, it is unclear if this would influence the dose to the heart because the primary analysis was done based on the initial treatment plans to 50.4 Gy.

A similar study was done by Fontanilla et al.,^(11)^ in which treatment plans were generated based on clinical landmarks as well as RTOG target volumes in the same 20 patients. They found that coverage of the target volumes was 74.0%‐96.0% when treating with clinically‐guided plans. In our analysis, coverage of many of the structures was higher than that reported in their series. The V95.0% for the chest wall and Ax 3 was 91.4% and 99.8%, respectively, in the Group I patients (see Appendix A), compared with 74.0% for the chest wall and 96.0% for Ax3 in the previous series. Our SCV dose was lower with V95 of 66.3%, compared to 84.0% in their series. Additionally, they found that the dose to the heart and lungs was higher with the target volume‐based plans, whereas we found that the low doses to the heart and lungs were lower with target‐based treatment planning. One possible reason for this discrepancy may be that in our analysis, the DVH parameters were generated from the treatment plans used to treat patients in both groups whereas, in the Fontanilla analysis, the target volume‐based plans were generated primarily as a dosimetric comparison. Therefore, more emphasis may have been placed in trying to minimize dose to the OARs in our cohort of patients. Our analysis suggests that the RTOG target volumes can be used to develop treatment plans as long as appropriate dose constraints are used for OARs. While dosimetric studies such as these support that the RTOG guidelines can be implemented safely, it is unclear if using these guidelines will improve oncologic outcomes as using traditional clinical planning has resulted in excellent outcomes with long‐term follow‐up.

## V. CONCLUSIONS

Overall, our study supports that target volume‐based treatment planning using the RTOG targets will not increase dose to surrounding tissues beyond clinically accepted limits, while there may be improvement in coverage to certain target volumes. However, when implementing target volume‐based planning, dose constraints to organs at risk must be carefully observed. Longer follow‐up will be needed to determine if using these target volumes will affect clinical outcomes, such as local control, regional control, and overall survival.

## ACKNOWLEDGMENTS

This work was supported in part by the National Institutes of Health (NIH) Grant No. 2T35AG029795‐06

Support for institutional review board approval was provided by the University of Chicago Comprehensive Cancer Center support grant P30 CA014599

## Appendix A: Mean coverage of target volumes in Group 1 vs. Group II for additional DVH parameters.

**Table:**

*Structure*	*Group I Clinically‐derived (mean)*	*Group II Target Volume‐based (mean)*	*p‐value*
CW (V95^a^)	91.4	93.2	0.31
SCV (V95)	66.3	87.3	0.0006
Ax 3^b^ (V95)	99.8	99.5	0.68

^a^ Vx refers to the volume of the target volume receiving x% of the dose (i.e., V95 refers to the volume receiving 95.0% of the prescription dose).

^b^ Ax3 means axillary lymph nodes, level 3.

CW = chest wall; SCV = supraclavicular lymph nodes.
